# Interpersonal stranger violence and American Muslims: an exploratory study of lived experiences and coping strategies

**DOI:** 10.1080/11287462.2019.1683934

**Published:** 2019-10-30

**Authors:** Priyanka Agrawal, Yousra Yusuf, Omrana Pasha, Shahmir H. Ali, Homayra Ziad, Adnan A. Hyder

**Affiliations:** a Department of International Health, Johns Hopkins Bloomberg School of Public Health, Baltimore, MD, USA; b Department of Population, Family and Reproductive Health, Johns Hopkins Bloomberg School of Public Health, Baltimore, MD, USA; c Department of Social and Behavioral Sciences, College of Global Public Health, New York University, New York, NY, USA; d Program in Islamic Studies, Kreiger School of Arts and Sciences, Johns Hopkins University, Baltimore, MD, USA; e George Washington University Milken Institute School of Public Health, Washington, DC, USA

**Keywords:** American Muslims, violence, coping strategies

## Abstract

Hate crimes in the United States have drastically increased since 2015, particularly for the American Muslim population. There was a 17% hike in hate crimes against American Muslims in 2017 compared with the previous year. The objectives of the study were to document the experiences of interpersonal stranger violence, coping strategies and recommendations by American Muslims. We applied qualitative research methods to conduct seven focus group discussions with 37 participants in the Maryland area, throughout 2017. There were reports of verbal abuse, discrimination (in schools, workplace, college campuses, airports, Visa offices), bullying and microaggression. Individuals were torn between the public anxieties of being Muslim and their private attachment to their religious identity. Despite reports of fear and uncertainty, individuals applied caution, positive religious coping, and encouraged family and community engagement to gain and provide support to each other. This study illustrates the consequences that the 2016 US presidential election and Islamophobic rhetoric had on American Muslims. Further research will elucidate the long-term impact on health outcomes of these behaviors.

## Introduction

The incidence of violent crimes has fallen significantly in the US over the last two decades. However, there has been an increase in the numbers since 2015 compared with previous years (Glaser & Strauss, 2017; Stuart et al., 2006). Hate crimes increased by 67% in 2015. (“^2015^ Hate Crime Statistics,”). Of all the hate crimes reported in 2015, one in five (1,354 out of 6,885) were due to religious bias. Specifically, anti-Muslim bias was reported in ∼22.5% cases.(Cordesman, 2017) The number of assaults against Muslims in the US increased in 2015 – 16 to higher than in 2001, the peak violence against Muslims following the twin tower attacks on 9/11.(“Violent crime in the U.S. – Statistics & facts”) The rhetoric surrounding the presidential election of 2015 saw an increase in anti-Muslim hate crimes by 17% in 2017 compared to the previous year.(Soltani, 2018)

The reported hate crimes have ranged from fatal and non-fatal assaults on Muslims and those perceived to be Muslim, and threats against and destruction of mosques and other property believed to belong to Muslims.(Levin & Grisham, 2016) These statistics, however, do not capture the true extent of such violence as more than 60 percent of violence perpetrated by strangers, friends and acquaintances goes unreported (“Hate Crime Victimization, 2004 - 2012 - Statistical Tables”, 2014). The rising anti-Islamic hostility often depicted by the media is unlikely to be accurately captured by existing enumeration mechanisms (Awad, 2010).

Violence is associated with various negative health outcomes and behaviors and varies by gender and socio-economic status.(Guyll, Matthews, & Bromberger, 2001; Sellers, Bonham, Neighbors, & Amell, 2009; Sims et al., 2015) Perceived discrimination is linked with poor health care-seeking behaviors (such as decrease in cancer screenings and poor diabetes management) and increase in smoking, alcohol consumption and substance abuse (Landrine & Klonoff, 1996; Ryan, Gee, & Griffith, 2007; Yen, Ragland, Greiner, & Fisher, 1999). However, victims of violence rely on social support, community engagement, and religious practices to cope with the after effects of violent situations (Coker et al., 2002). Although studies exist on the health effects of discrimination and violence, there is a dearth of research on the effects of violence in the current political climate on American Muslim communities (Samari, Alcalá, & Sharif, 2018).

The reported increase in anti-Muslim hostility and research indicating an impact on health behaviors associated with discrimination and violence lead to an exploratory study to document the nature, types and forms of interpersonal stranger violence reported by American Muslims. In addition, the study aimed to understand the coping strategies imbibed by and health and social behavior changes implemented by American Muslims to adapt to changing environments. The hope is to stimulate scientific discourse on this increasingly reported issue comprising a public health hazard for American Muslims.

## Methods

For the purpose of this study, the World Health Organization’s (WHO) definition of interpersonal violence –

violence between individuals, and subdivided into intimate partner violence and community violence … and community violence is broken down into ***acquaintance and stranger violence*** and can include youth violence, assault by strangers, violence related to property and violence in workplaces and other institutions

was used to describe violence (Krug, Mercy, Dahlberg, & Zwi, 2002, emphasis in the original). This study focused on *acquaintance and stranger violence* against American Muslims in the US.

### Participants

We conducted informal meetups with religious leaders (imams), senior representatives of community-based organizations, activists and Muslim student associations at various universities in the Maryland area. We provided a general overview of the project and gauged their interest in assisting with recruitment of participants. Subsequently, we conducted *seven* focus group discussions (FGD).

The study sample included 37 participants (females = 24; males = 13). (Table 1) All participants self-identified as Muslims and had been residing in the US for at least a year prior to the time of discussion. The focus groups comprised of individuals across age, gender, race and socio-economic status. Participants were between 18 and 64 years of age and identified as African American, South Asian, African and Arab. The sample had students, teachers, professors, activists, healthcare providers and faith leaders. Due to the exploratory nature and sensitivity of the study, detailed demographic characteristics of the participants were not collected.

**Table 1.** Socio-demographic characteristics of participants in focus group disucssions.

Focus group	Location	# of participants (Females, Males)	Average age (years)	Professions represented	Duration (minutes)
1	University	5 (4 F, 1 M)	35	Student, Doctor, Researcher	52
2	University	4 (2 F, 2 M)	19	Students	48
3	Home of a participant	4 (3 F, 1 M)	48	Activists	93
4	University	6 (3 F, 3 F)	35	Students, teachers, staff	62
5	Home of a participant	9 (5 F, 4 M)	53	Physicians, University staff	87
6	Community-based organization	5 (5 F)	32	Staff, activists, community members	108
7	University	4 (2 F, 2 M)	45	Students, Faith leaders, staff	110

### Process

Based on the study objectives, semi-structured FGD guides with questions around experiences of interpersonal violence and mechanisms established at individual and community levels to cope with violent incidents were developed. (Annex 1) Each FGD was facilitated by two members of the research team and were conducted in English. The discussions occurred in convenient locations for participants – university settings, offices, homes of participants, and had an average of 5 participants. (Table 1) The discussions were audio-recorded and hand-written notes were also kept after written informed consent was obtained from all participants. The FGDs ranged from 48 minutes to 110 minutes in duration (average time: 80 minutes).

### Data analysis

The primary goal of our study was to initiate a line of research on a vulnerable population that has been under-researched. First, the seven audiotaped conversations from the FGDs were transcribed. However, due to technical error, full transcripts from six FGDs and a portion of the seventh FGD (assisted with handwritten notes) were included in the analysis. All participant identifiers were redacted during the transcription process.

Pre-defined, broad, thematic categories were used to construct a list of *a priori* codes with definitions. The broad themes were (1) types of interpersonal stranger violence, (2) coping mechanisms, (3) media coverage, and (4) future actions. We imported the transcribed FGDs into the ATLAS.ti qualitative software program to conduct an in-depth content analysis of one transcript (Smit, 2002). Two coders who were part of the FGDs, systematically read through the transcript to identify additional emergent thematic categories. The coders then developed additional codes and refined the coding framework, that was reviewed by a third research team member. Thus, a mixed approach of thematic and grounded theory was applied to guide the analytic process (Glaser & Strauss, 2017; Strauss & Corbin, 1990).

This was followed by a discussion of coding for quality assurance purposes and a second round of coding of the transcript to detect variations. Quotes for each code were examined and matrices and memos were used to organize and examine the information for patterns to develop emerging interpretations. The codebook was refined until no new thematic relationships were identified in the transcript, and a consensus was attained on codes to include their structural relationships. The researchers (three practicing Muslims and a South-East Asian non-practicing Hindu, all immigrants and either naturalized citizens of the US or residing in the US for education and employment) considered their own, as well as each other’s lived and past experiences as it related to the topic of violence.

Subsequent six transcribed texts were coded with strictly defined parameters. Within the aforementioned four broad categories, codes were categorized and sub-themes were sorted into more narrow constructs, concepts, and categories that allowed for data interpretation.

Ethical approval for our study was obtained from the Institutional Review Board of the Johns Hopkins Bloomberg School of Public Health.

## Results

### Experiences of interpersonal stranger violence

Experiences of violence spanned across various forms – mental, physical, verbal and structural. Discriminatory and biased behavior were reported in a range of settings – schools, college campuses, airport, workplaces, malls and in the community – by individuals across age and ethnic backgrounds. Media was named as a source of perpetuating bias.

The media coverage of the 2016 presidential election and the widespread anti-Islam rhetoric made respondents feel unsafe. Children identified as Muslims were bullied and discriminated by fellow students and teachers, including those from other communities of color, and were ignored post-election by friends at school. Non-existent support systems in school settings as well as conflicted relationships affected children and their parents, so much so that some children were wary of being associated with Islam in the public domain:

she’s interesting because she went from, you know I’m a Muslim right – back then, to now she’s like, well – I’m not ‘that kind of Muslim’ […] and I think she’s tried to distance herself from Islam in general. – a South-East Asian female (25–35 years, social activist)

Participants reported similar incidents in colleges where students did not feel confident about the system supporting them (if need arose) and had set up their own codes of communication to stay connected. Diversity departments at universities faced barriers in facilitating interactions between faculty, staff and students to create comfortable learning spaces. Microaggression was seen within academic settings. (Table 2) An African American diversity and inclusion officer at a university with a broad diaspora of local and international students shared her experiences working with faculty and staff:

I facilitated conversations with staff, um, who have […] expressed discomfort and fear of Arab students who are upset, if they had something wrong that they were addressing, American faculties or staff members about and they were visually upset - they would call security very quickly. It’s a very quick response to get the student out of the room, restrained, and removed because they feel unsafe - and if they say something in Arabic while they’re upset, it is a whole another level of discomfort. – African American female (25–35 years, educator)

**Table 2.** Key emergent themes and findings from the study.

Broad themes	Topics	Sub-themes/Quotes
Experiences of interpersonal violence	Bullying	– Peers bullying fellow classmates in school settings A participant mentioned: “my two nephews have transferred school because they have [been] picked on so much, um – they have transferred from one elementary school from another because they’re Muslim. So – yeah, to like my kindergartener and little grade you’re a terrorist, you’re here to kill us, you’re gonna bomb us, we don’t want to play with you – totally ostracizing them, teachers picking on them too” – South Asian Male (34–45 yrs, activist)
	Discrimination	– Educational Institutions A student’s encounter with a white conservative professor left her feeling very silenced: “it was the election season, um, he I had no idea that he was – very – big Trump supporter. His classes were predominantly white, it was me and maybe – one or two other minorities in the classroom, which was the least out of every class I’ve ever taken … it was terrible. And he would make comments or say things about sect, it’s not very geared towards me, or anyone else, I was just imagining this, until one of the other minorities in the class pulled me aside and was like, do you notice this too? Ok, this is not just me, um, when we both started making comments back towards him, and he would take points off of things from us, and it was – a whole big thing … [we felt] Extremely silenced. We realized that he couldn’t stop his thinking, we were tired of being silenced by him.” – African Female (15–25 years, student) – Workplace settings An activist who traveled to present her organization’s work shared: “I’ve had people – come up to me straight and say, if you were wearing a hijab during that presentation, I would not have been able to hear anything you said … . And I said thank you for saying that thank you for owning that, and now I’d like you think about – what that says about the way that you perceive of this one piece of cloth – that would have been the difference between you actually listening to me and not listening to me, meaning humanizing me and dehumanizing me, basically.” – African American Female (35–45 yrs, activist) – Within families – Airports, supermarkets, visa offices One person stated: “I don’t know what my legal rights, what, limits are, so I just felt, whenever you’re having a problem, just whatever, just do it, don’t argue.” – Asian male (45–55 yrs, physician)
	Verbal abuse	– Derogatory remarks – Insults – Hate language
	Property damage	– Vandalism of mosques, Muslim owned property
Media coverage of interpersonal violence	Impact on American Muslims	– Reports of fear, discomfort and anxiety A participant reported: “That’s in the back of your mind, so that’s created an environment to make people uncomfortable – especially, because, what you hear in the news now is the racist groups have been very vocal, what they’ve done. And police officers, it’s also true, I think that we’re not sure if the law enforcement is on your side now – what the discrimination against African Americans, that tase or kill – and they get away with it, so it’s giving that environment which is making you uncomfortable, and – unequivocally, no question, it is starting from the top. If the leader of the nation … is setting that, mindset, it filters down, and it’s sanctioning this kind of behavior … ” – African American male (45–55 yrs, faith leader) One parent emotionally recounted her interaction with her daughter the day after the 2016 US Presidential election results were announced:“ [Of]‘Course we all were expecting Hillary would win, and so, when she [daughter] woke up in the morning, the first thing – she asked was – did Hillary win? And I said, no, and she starts crying. She started crying and [participant tearing up*], later that day she was packing her backpack – full of all these things that she would take to an internment camp if they ever came to get us [Muslims].” – Asian Female (35–45 yrs, social activist)
Safety	Geographic Location and time of the day	A participant reported: “definitely when I kind of go outside of the city … I sometimes feel a little bit less safe, in certain situations, so I wouldn’t go a certain places and try to stay low in those places based on the context.” – Asian Female (25–35 yrs, physician) Another female participant mentioned: “So mostly I mean really driving to more of rural Pennsylvania, those compared to Virginia, I don’t feel as safer. So I try not to flaunt the fact that I am a Muslim very much because specially seeing trump signs and all those kinds of things make me feel uncomfortable and if someone knew certain things about me, they either wouldn’t like it or would actually do something that could be harmful.” – Asian female (25–35 yrs, physician)
	Self-identifying label	One woman shared her feelings that resonated with other participants: “I feel safe mostly. I have lived in very diverse communities since 2010 and a lot has happened, the 9/11 happened, and a lot of the anti-Muslim talks started in the United States and Umm have gotten worse. (long pause) I think that I have not had any personal problems, I don’t wear a hijab, and I think that it sort of allows me to slip under the radar. Everybody who knows me knows I am a Muslim but if you don’t know me, you wouldn’t necessarily know that I am a Muslim. I don’t go around saying that, I don’t label myself, so I think that it sometimes makes it easier for me.”(45–55 yrs, physician) A woman activist shared her dilemma with practicing Islam after the last presidential election, which also reflect the confusion the general population has about color and ethnicity: “I am a community activist and – um – because I don’t wear hijab, so then a lot of things don’t happen to me but uh – because I look South Asian, so they don’t know I’m [a] practicing Muslim. The, uh, before Trump when my kids were very busy after school I usually pray one or two namaz in my car, so after that my husband said don’t do that because somebody can know something when you are praying in the car, so that becomes like, scary thing, even though I pray, because uh – I have to sit two three hours with them, so – I feel like somebody is going to knock on my thing [car window] or throw something on me.” – African American female (25–35 yrs, activist)
Coping strategies	Individual-level	– Vigilance and caution A participant reported: “I think one thing I may be not speaking up as much depending on the audience about certain things when it comes to people talking about either racial kinds of things or politics even depending on the audience I don’t feel as comfortable. I think that’s less, I don’t think its necessarily about being a Muslim, but I think its just more about realizing that there are lot more people that feel a certain way that’s different in real life.” – Asian female (15–25 yrs, student) – Emphasis on Attire and external appearance – Positive religious coping
	Community level	– Community engagement A participant beautifully explained the power of healing from community support:“So, there was a gathering at – Maryland for Hillary at […]asked people to get up and speak their truth, so I got up and told what I’ve just told you, how [.] had felt, and – I’ve never felt such an outpouring of love, from a whole group of strangers in a room, and I will never forget, never forget what [.] said to me, he gave me a hug, and he said, the Democrats will always have your back. That’s what he said to me that night, we will always have your back, you have there is no reason for you to feel – this unsafe, it’s not going to happen, what [.]’s fear will not happen.” – South Asian female (45–55 yrs, Activist) – Reaching out to family, friends, neighbors and acquaintances. – Safe Spaces such as Open house for children, adolescents, Muslim and interfaith community members One participant stated:“on a personal level – you know, I think somebody mentioned that we have these events and bringing other friends, but, our neighbors and our immediate community have more right on us, on our hospitality, on our time, and on our efforts, but then, our immediate neighbors, we should be helpful, we should be show them because everyone has problems, not just Muslims, we can convince, we can prove to our neighbors, our community that we are good citizens, we are law abiding people, and then make that work for us rather than against us, and often we identify as a Muslim, certainly is, I’m proud to be Muslim, but, that should not be the first thing that comes into people’s minds when they see us.” – South Asian Male (55–65 yrs, physician) – Self-defense and emergency preparedness training. – Community resources One community activist highlighted the need to work from within the community to make resources available for the community and others in emergent situations: “if there is a deportation crisis, do you have a list that you can blast and say here are the resources you need, here is what we can provide, and here are the networks you know, like with – when DACA happened –– I could connect those so quickly because of Catholic Charities and because of the organizations that exist. We don’t have that with the Muslim community, when I called to find whose doing this – there was nothing, there was no structure in which to get the Muslims the information that they need. But there was for other communities, and it made me realize we are so bothered with rules and regulations in our communities – that we need to break those down and be like we are Muslim – and we need to come together and we need to be intersectional and, within our own communities, and we need to raise our voices, and how do we get that, to that point?” – South Asian female (35–45 yrs, activist) – Engagement with interfaith organizations, Muslim organizations, Charities, public office.

Incidents of veiled prejudice were common in workplace settings. (Table 2) For some who did not carry a self-identifying label of a Muslim, such as a hijab, skull cap or long beard, it was easier to navigate their workplace and stay away from discriminatory advances. Muslims were concerned about having to give up some (or all) of their Muslim identity to advance professionally.

No participant reported being physically assaulted or had their property damaged or vandalized, but media coverage of such events had instilled fear, discomfort and hurt among all. However, participants reported facing derogatory remarks, insults and hate language from family members (of another faith), acquaintances, customers, and community members. Incidents with perpetrators assuming collective guilt and apparent double standards in the face of global events perpetrated by alleged Muslims also came across, as reflected by a student:

I want to mention something I feel there – if there is any crime in United States, Muslim are a part of it – they, I guess, the US government – make the crime belong to us as, but for example the white American did – say “Domestic Terrorism”, then maybe he is having a mental problem, but, why the other Muslim maybe they have a mental problem. – Asian male (15–24 years, student)

One elderly participant, amused and saddened at the same time, about the perceptions of communities towards Muslims and Muslim owned property noted:

I would say 6 months or a year ago, there was this group, I think somewhere out of Pennsylvania. There’s a mosque here in [..] county and they found out about that mosque, found out on social media, and they said that we’re going to come in and, you know, protest outside the mosque. These guys were from outside, they had no idea of what this mosque does, good or bad. But somebody just riled them up that oh ‘here’s a mosque, you need to go and protest.’ But my point is that this is a group of people who drove for maybe an hour or two hours or three hours, just to come out and protest at this mosque, that they have never seen in their life before, and they may never see again- South-East Asian female (45–55 years, social activist)

There were reports of discrimination at airports – additional screenings, luggage checks, and sharing passwords of electronic devices with customs officials. Participants were conflicted within themselves on whether some of these actions were “legal” but were not comfortable to resist or question the authority (Table 2).

### Perceived safety

Majority of participants expressed that personal safety and well-being were closely linked with the geographic location of public space. (Table 2) Participants generally felt safer in diverse communities but were not as comfortable in predominantly white, Trump supportive communities. (Table 2) Men reported being more comfortable in their surroundings and felt that women, children and the elderly were more vulnerable. The stereotyping and Islamophobic rhetoric in media has instilled fear and an inherent deep-rooted discomfort among participants and their acquaintances. (Table 2) A participant’s comment touched on discomfort heightened by disparities and increased political tension:

It was between the republican convention and the election. So I was … in a certain part of Ohio which was economically very depressed, they had gone through all kinds of issues, and just driving through these little towns in south-eastern Ohio you could feel that the people are hurting,. And um, and one day I had to, I had to stop and get gas at a gas station. And um, the very, you know as soon as I pulled in I knew that – this is a very uncomfortable situation. I had pumped gas, went in, and that whole encounter, you know, lasted maybe a couple of minutes but it was extremely uncomfortable. – A South-East Asian Male (35–45 years, physician)

Participants reported that people related the color of skin with Muslim identity, or practicing Islam with discomfort. Most participants were uncomfortable sharing their views (including political) in public spaces due to the perceived intolerance and conflicting viewpoints in communities. Some participants noted that their practice of Islam, as in praying in the workplace, identifying as a Muslim, or wearing the hijab, fostered a sense of uneasiness among others. Reports of discrimination highlighted the confusion and dilemma around color and ethnicity (Table 2).

### Coping strategies

Participants reported exercising caution and vigilance when not in the safety of their homes, and paid attention to what they were wearing or how to open they were in sharing their thoughts in public. Some parents mentioned having pep talks with their school-aged children on sharing any and all issues they might face in school settings with parents. (Table 2)

Some respondents got more involved within their communities, local Muslim councils, reaching out and showing solidarity to their Muslim brothers and sisters. Support from non-Muslim neighbors, co-workers, and bystanders also helped some individuals focus on the positives, and friends were looking out for their Muslim friends on campus grounds. A student recalled an encounter with a teacher, who despite her republican and conservative ideologies, openly supported the student’s ideas. A particular school provided “safe spaces” for children and teachers observing Ramadan to pray, despite having a predominantly non-Muslim, mixed-faith leadership.

Conflicting political views forced two participants to distance themselves from family and known acquaintances. A few women participants reported withdrawing their positions and engagement from neighborhood associations and community groups that fostered an environment of neglect, exclusion and overt discomfort towards Muslims. Participants were selective in their intake of news and emphasized on checking facts before processing news segments. A participant in an interfaith family noted:

We haven’t felt threatened by somebody, but my husband is American and he is a Muslim, his family is not and they never had a problem with me before, they don’t have a problem with me being a Muslim, but I have felt threatened by their support of Trump. When I wrote to my mother in law that it bothers me, she didn’t respond. So I didn’t contact her again, all that summer. You know, that was incredibly hurtful, I think, not threatening.- Female (45–55 years, researcher)

An activist helped organize self-defense classes for women in response to hijab snatchings in university settings. Many participants sought self-reflection and connection with Islam to strengthen their mental health and educate themselves to spread knowledge and counter the hoax anti-Muslim information. Younger participants were revisiting their Muslim identity and using the energy to educate and to create safe opportunities to interact with other communities. They also exercised caution in trusting news on social media platforms although they relied heavily on those platforms than major news outlets. One college educator, with more than 40 years of experience working with young children and adolescents noted:

I think that there’s part of it is kind of – increased pride in a Muslim identity –it’s just that increased, I guess pride in the identity of being Muslim, over culture, over race, over – not despite them, but just in a way that would.. we’re all, we’re all Muslim, and being able to show that to one another person foremost, I think it’s more than just representing who Muslims are to non-Muslims- an African American male (65 years and above, educator)

Participants working in the education and civil rights sectors advocated to improve efforts on hate crime reporting procedures. Some participants discussed the need to spread awareness and education on Islam and Muslims to the broader community to stimulate acceptance and cultural sensitivity. Participants emphatically mentioned the importance of engaging in community work – volunteering for charitable causes, increasing involvement in broader society or participating in politics. Suggestions were made for families to stay connected during times of crisis, be role models for their younger generations, and encourage young children to learn and educate their peers in the values of Islam. There was a need for accessible and accepting community spaces – for all members of the society. Emphasis was placed on Muslim communities breaking out of silos, looking beyond race, class and status, and engaging with neighbors and the immediate community. The idea was to create stable amalgamations of cultures, backgrounds and perspectives. (Table 2)

## Discussion

Our research had three goals: (a) to document the types and nature of interpersonal stranger violence faced by American Muslims in the US, (b) to recognize coping strategies and (c) to identify recommendations to improve social and health behaviors in response to violence. Our findings on the nature and types of interpersonal violence are similar to studies on heightened anti-Islam movement after the September 11, 2001 attacks. Similar incidents of physical assault, bias and discrimination, derogatory remarks, verbal abuse, and property damage had been reported (Abu-Raiya, Pargament, & Mahoney, 2011; Awad, 2010; Disha, Cavendish, & King, 2011; Hodge, Zidan, & Husain, 2017; Wright, Wallace, Bailey, & Hyde, 2013). This study re-emphasizes the complex nature of impact violence has on individuals and communities – physical, mental, and economic (Carlson, 2005). As mentioned in earlier studies, interpersonal violence generated fear, anxiety, stress and depression among American Muslims (Jalalzai, 2011; Samari et al., 2018). The rise in Islamophobia – in direct response to the anti-Muslim rhetoric during the 2016 presidential campaign and biased media coverage – has had a negative impact on individuals.

Violence was reported in all settings – neighborhoods, schools, college campuses, workplaces – across all age groups and gender. There were second-hand reports of children and adolescents being subjected to bullying, neglect and discrimination from friends, classmates and teachers. Childhood exposure to bullying and discrimination can lead to distress, adjustment issues, and poor long-term outcomes for both victims and perpetrators (Bernstein & Watson, 1997; Sigurdson, Undheim, Wallander, Lydersen, & Sund, 2015). Facing negativity from peers due to religious identity can affect spirituality and faith in one’s religion, as was noted in this study. Spiritual development can have an important role in human development and be a source of strength for individuals (Benson, Roehlkepartain, & Rude, 2003).

Individuals shared mixed perceptions of safety in reponse to interpersonal stranger violence, unlike after the 9/11 attacks, where most felt unsafe (Cordesman, 2017). The surroundings played a huge role in perceived safety for oneself and families. Individuals were torn between the public anxieties of being Muslims and their private attachment to their religious identity. Positive religious coping mechanisms were widely applied to feel at peace (Abu-Raiya et al., 2011; Rodriguez Mosquera, Khan, & Selya, 2013). Of note, the younger generation used positive religious coping to educate others and create safe spaces for conversations. Older individuals were precautionary in their interactions with community and believed that the current state of events would mellow down. However, the need to interact with others to create safe, stable, comfortable and heterogenous communities was expressed on multiple occasions.

As has been seen with other forms of interpersonal violence cases, emotional avoidance of perpetrators and limited expression of thoughts were exercised by some participants (Tull, Jakupcak, Paulson, & Gratz, 2007). These are commonly applied in varying extents by people who suffer from post-traumatic stress disorder. However, most participants reported getting involved with families, friends, acquaintances and communities, in general, to look for and provide support (Zink, Jacobson Jr, Pabst, Regan, & Fisher, 2006). Involvement ranged from non-structured interactions like keeping in touch, to more structured interactions such as participating in Muslim councils, interfaith organizations, charities and politics. Studies have shown reduction in symptoms of post-traumatic stress disorders with family and social support in situations of community violence (Scarpa, Haden, & Hurley, 2006).

The data provides evidence that certain strategies could be helpful for Muslims in the US to cope with and nurture their lives in response to faith-based violence. Self-defense training and emergency preparedness training can help individuals protect themselves from physical assaults. These also instill self-belief and confidence. Individuals could also be selective in their choices of media and fact check information. Positive religious coping, spirituality and faith-based approaches such as confirmation of beliefs, engagement in religious behaviors, and support from faith communities are protective for interpersonal violence (Ahrens, Abeling, Ahmad, & Hinman, 2010).

This exploratory study has multiple implications for future research on interpersonal stranger violence towards American Muslims. More research is needed to examine, in greater depth and on a larger sample, the short and long-term consequences of interpersonal violence perpetrated on minority populations. The knowledge could help minority populations and those working to improve health and well-being for such populations, to design policies and programs to help victims of violence.

Additionally, an investigation of the effects of segregation within minority populations will provide insights into the dynamics of race, status, class, and color within the Muslim community – the strengths and weaknesses within the population could inform program and policy design for the population. Even though a minority population is studied with a single lens, it is important to acknowledge the heterogeneity within a minority population. Further research is needed to (1) understand coping strategies used by diverse sections of the Muslim community to violent incidents, and (2) assess impact of individual-level and structural-level Islamophobia on American Muslims.

There are several limitations to this study. The sample was small and regional given the recruitment methods, so its findings are not generalizable to all American Muslims living in the US. Since the study looked at experiences of violence, it is possible that people who volunteered to participate may have experienced violence or had strong opinions around this topic. Additionally, the sample was not heterogeneously distributed across racial, ethnic and socio-economic backgrounds, thus did not represent the full diversity of American Muslims.

In conclusion, this study illustrates the consequences that the 2016 US presidential election and Islamophobic rhetoric had on American Muslims, who report a higher level of physical, mental, verbal, and structural violence in the community. Despite reports of fear and uncertainty in the wake of increased violence, communities have identified and engaged in supportive individual, family and community-oriented behaviors to cope with assaults on the American Muslim population. Further research will elucidate how such behaviors can impact health outcomes in the long-term.
